# Use of Accelerometer Activity Monitors to Detect Changes in Pruritic Behaviors: Interim Clinical Data on 6 Dogs

**DOI:** 10.3390/s18010249

**Published:** 2018-01-16

**Authors:** Susan M. Wernimont, Robin J. Thompson, Scott L. Mickelsen, Spencer C. Smith, Isabella C. Alvarenga, Kathy L. Gross

**Affiliations:** 1 Pet Nutrition Center, Hill’s Pet Nutrition, Inc., Topeka, KS 66617, USA; scott_mickelsen@hillspet.com (S.L.M.); kathy_gross@hillspet.com (K.L.G.); 2 Open Lab, Newcastle University, Newcastle-upon-Tyne NE1 7RU, UK; r.j.thompson3@newcastle.ac.uk; 3 Department of Grain Science and Industry, Kansas State University, Manhattan, KS 66506, USA; spence5@k-state.edu (S.C.S.); isacorsato@ksu.edu (I.C.A.)

**Keywords:** wearable sensor, activity monitor, dermatitis, scratching, shaking, dermatology, pruritus, accelerometer, nutrition

## Abstract

Veterinarians and pet owners have limited ability to assess pruritic behaviors in dogs. This pilot study assessed the capacity of the Vetrax^®^ triaxial accelerometer to measure these behaviors in six dogs with pruritus likely due to environmental allergens. Dogs wore the activity monitor for two weeks while consuming their usual pet food (baseline), then for eight weeks while consuming a veterinary-exclusive pet food for dogs with suspected non-food-related skin conditions (Hill’s Prescription Diet^®^ Derm Defense^TM^ Canine dry food). Veterinarians and owners completed questionnaires during baseline, phase 1 (days 1–28) and phase 2 (days 29–56) without knowledge of the activity data. Continuous 3-axis accelerometer data was processed using proprietary behavior recognition algorithms and analyzed using general linear mixed models with false discovery rate-adjusted *p* values. Veterinarian-assessed overall clinical signs of pruritus were significantly predicted by scratching (β 0.176, *p* = 0.008), head shaking (β 0.197, *p* < 0.001) and sleep quality (β −0.154, *p* < 0.001), while owner-assessed quality of life was significantly predicted by scratching (β −0.103, *p* = 0.013) and head shaking (β −0.146, *p* < 0.001). Among dogs exhibiting pruritus signs eating the veterinary-exclusive food, the Vetrax^®^ sensor provided an objective assessment of clinically relevant pruritic behaviors that agreed with owner and veterinarian reports.

## 1. Introduction

Canine atopic dermatitis (AD) is a condition commonly seen in veterinary practices [1,2,3], and is defined as “a genetically predisposed inflammatory and pruritic allergic skin disease with characteristic clinical features associated with IgE antibodies most commonly directed against environmental allergens” [4]. AD can have a seasonal or non-seasonal presentation, with non-seasonal being more common [5]. Diagnosis is based on clinical examination by a veterinarian; characteristic features are the presence of pruritus along with skin lesions around the mouth, ears and eyes and on the paws, abdomen and perineum [5,6]. Current recommendations on the treatment of atopic dermatitis encompass both lifestyle and pharmaceutical approaches, including elimination of allergens, modifications to coat hygiene and care, topical shampoos, sprays, lotions and medications such as glucocorticoids, oclacitinib, cyclosporine and injectable recombinant interferons [7]. 

Like the initial diagnosis, assessments of AD severity and decisions about management have traditionally been based on signs observed by veterinarians or reported by pet owners, particularly pruritic signs [8]. This requires either close monitoring of the patient or examination of skin abrasions caused by scratching. However, both approaches are time consuming and prone to inaccuracy and subjectivity. Since veterinarians and owners are limited in their ability to accurately assess pruritic behaviors, attempts to assess the degree and severity of pruritus have been made using automated approaches. Behaviors of healthy and pruritic dogs have been distinguished using activity monitors [9]. Similarly, correlations were seen between data from ActiCal^®^, an activity monitor that measures a generic activity level, and observed nocturnal pruritus time [10]. Notably, both of these examples rely upon the use of general activity measurements made using sensing platforms unable to distinguish between specific forms of activity or behaviors.

In order to recognize and quantify individual behavior patterns, more complex systems such as triaxial accelerometer technology are required. The Vetrax^®^ behavior monitor (AGL Technology, Norcross, GA, USA) employs proprietary behavior recognition algorithms to interpret triaxial accelerometer signals in terms of the behaviors being expressed. Video validation of such an approach was recently described; the Vetrax^®^ system reports scratching and head shaking with an accuracy of 99.24% and 99.56%, respectively [11]. This allows for targeted monitoring of pruritic behaviors, including scratching, head shaking and sleep quality, all of which may be altered in canine dermatitis. In the present report, we provide interim data from an ongoing clinical trial assessing the capacity of the Vetrax^®^ activity monitor to quantify the frequency and duration of pruritus-related behaviors (scratching, head shaking and sleep quality) in dogs with pruritus likely due to environmental allergens, to monitor changes in these behaviors in response to a nutrition intervention and to compare them with veterinary and pet owner evaluations. 

## 2. Materials and Methods

### 2.1. Study Design and Animal Selection

Six client-owned dogs who, after ruling out other pruritic skin conditions (including flea allergy, contact dermatitis and sarcoptic mange), were diagnosed with pruritus likely due to a seasonal or nonseasonal allergy unrelated to food, were enrolled at three privately owned veterinary clinics in northeast Kansas (Shawnee, KS; Lawrence, KS; and Topeka, KS, USA). While not definitive, the pruritic diagnosis used in this study reflects the population of dogs likely to receive Hill’s Prescription Diet^®^ Derm Defense^TM^ Canine dry food (Hill’s Pet Nutrition Inc., Topeka, KS, USA). Dogs remained in their homes and wore a collar-mounted Vetrax^®^ activity monitor throughout the study; activity monitors collected acceleration data which was aggregated to fifteen minute increments throughout the day and processed using proprietary behavior recognition algorithms. At the first clinic visit (designated Day –14, as it occurred 2 weeks prior to the Day 0 visit), pet owners provided written consent for their dog to participate in the study, investigators evaluated each dog for study eligibility and an activity monitor was dispensed. During the two-week baseline period (Day –14 to Day 0), dogs consumed their usual pet food while wearing the activity monitor. On Day 0, eligible dogs continued to wear the activity monitor and were fed Hill’s^®^ Prescription Diet^®^ Derm Defense^TM^ Canine dry food for the following eight weeks. Owners and veterinarians completed questionnaires at the Day 28 and Day 56 visits to describe the condition and pruritic signs of each dog for each study phase including baseline, phase 1 (Days 1 to 28) and phase 2 (Days 29 to 56) without knowledge of the activity monitor data. A window of ±3 days was allowed to facilitate scheduling of the Day 0, Day 28 and Day 56 visits. 

All aspects of the study were conducted in compliance with the Hill’s Pet Nutrition Global Animal Welfare Policy, and the study design was approved by the Hill’s Institutional Animal Care and Use Committee and Animal Welfare Committee (CP701). 

### 2.2. Study Population

Dogs that met the following inclusion criteria were considered eligible for study enrollment: at least one year of age; consuming at least 75% of food intake as dry dog food at the time of enrollment; in investigator’s assessment, likely to complete an eight-week feeding period with the therapeutic food and a ten-week data collection period with the wearable activity monitor; owner had a functioning Wi-Fi network in their home and agreed to use a Wi-Fi enhanced wearable activity monitor to record their dog’s behaviors; owner agreed to hold constant the regimen for any medications, treatments and/or supplements for the target condition until at least study Day 28; dog received a clinical diagnosis of pruritus due to a seasonal or non-seasonal allergy unrelated to food, ectoparasites or contact with materials; at the time of enrollment, dog exhibited frequent shaking or scratching or licking of at least one area including the face, paws, distal extremities, elbows or ventrum; and dog successfully completed a two-week baseline data collection period using the wearable activity monitor. Dogs from single-dog households were preferred. All medications and topical treatments were allowed at study entry. Exclusion criteria were: history of a major concurrent systemic disease or condition, including but not limited to cancer, liver disease, diabetes mellitus, hypothyroidism, hyperadrenocorticism, seizures, vomiting, diarrhea, kidney disease and/or arthritis; receipt of any therapeutic dog foods in the twelve weeks prior to enrollment; Body Fat Index (BFI) <20; illness or need for hospitalization for underlying disease condition at the time of enrollment; dehydration and need for intravenous fluids; active infectious disease including but not limited to pneumonia or gastroenteritis; history of an unknown illness or a current illness that was not likely to obtain a diagnosis; participation in other clinical trials within the last six months; unwilling or unable to exclusively consume the study food; expected to be boarded at any point during the study period or living partially or exclusively outdoors. 

### 2.3. Clinical Outcome Measures

Study forms completed at each visit are summarized in Table 1. At the Day –14 visit, the first part of the eligibility checklist was completed, a physical exam was performed and medical and dietary history were obtained. During the Day 0 visit, the second part of the eligibility checklist was completed and eligible dogs were enrolled in the study. Dogs who were enrolled in the study received a physical exam which included BFI scoring (visual scale 20–70). Both veterinarians and pet owners completed questionnaires documenting signs of the animal’s condition, including a pet owner-completed Quality of Life assessment (Veterinary and Pet Owner Evaluations, visual analog scale; Pet Owner Quality of Life assessment, segmented numeric visual analog scale). Subsequent visits involved a physical exam and questionnaires documenting signs of the animal’s condition, including a pet owner-completed Quality of Life assessment (Veterinary and Pet Owner Evaluations, visual analog scale; Pet Owner Quality of Life assessment, segmented numeric visual analog scale), similar to those completed at the Day 0 visit. The results from the overall scores of these questionnaires are reported in this interim analysis; owner-assessed ear and skin scratching are also reported in the supplemental data. Questions considered part of the overall scores for veterinarians included questions on overall skin quality, overall coat quality and overall clinical signs associated with the condition. Questions considered part of the overall scores for owners included questions on the overall condition of the dog’s skin and haircoat, how disruptive the dog’s skin condition is to you or your family, and the dog’s quality of life. Additional questions on specific dimensions of the pruritus-related condition were also asked. Responses to veterinarian and owner questionnaires were assessed using a visual analog scale. Visual analog scales have previously been used to capture patient-reported outcomes and proxy-reported outcomes in pruritus studies [12,13]. Here, the visual analog scale consisted of a straight line with the extremes marked. Respondents were asked to draw a vertical line representing the point on the line corresponding to their response to the question. Responses were subsequently converted to a 100-point scale with 100 corresponding to the most severe signs and 0 corresponding to the least severe signs. Quality of life was assessed on a segmented numeric version of the visual analog scale in which the owner selected a whole number (1–10 integers) corresponding to their assessment of the dog’s quality of life, with 10 corresponding to excellent. During the Day 28 and 56 visits, owners were also asked to complete questions related to their opinions of food consumption, food acceptance, food aroma and stool consistency. Food consumption was reported as a percent. Food acceptance and food aroma were assessed by pet owners on a visual analog scale similar to those described above; responses were subsequently converted to a 100-point scale, with 100 representing the greatest acceptance and strongest aroma, respectively. Pet owners reported fecal scores on a segmented numeric version of the visual analog scale in which the owner graded the fecal consistency from 1–5, with 1 as liquid and 5 as firm.

Protocol deviations, changes to medications and adverse events (AE) were recorded as they occurred during the study. AE were defined as any observations in the dogs that were unfavorable and unintended, whether or not they were considered to be related to the study food. 

Daily information on temperature, number of daylight hours and humidity was obtained from https://www.wunderground.com/weather/us/mo/kansas-city; daily pollen and spore counts were reported from a single location in Kansas City, MO, USA. 

### 2.4. Wearable Activity Monitors

Vetrax^®^ activity monitors are small, lightweight devices that attach to a collar on the ventral side of the neck of each study subject. The monitor collected high frequency 3-axis accelerometer data on each dog throughout the study period. This collected data was uploaded regularly via Wi-Fi to the Vetrax^®^ cloud for behavior algorithm processing. The Vetrax^®^ system employs machine learning approaches for activity recognition based on the accelerometer signal produced by the sensor. Each sample recorded by the sensor is composed of three readings, one for each spatial dimension which, when considered together, describe the changes in velocity of the sensor over time. Acceleration readings produced by the sensor have distinct characteristics dependent on the behavior being exhibited. The proprietary Vetrax^®^ algorithm is able to distinguish between these behaviors through identification of these characteristics. While there are cases in which the algorithm may produce both false negative, and false positive classifications, the rates at which these occur are low. A thorough evaluation of the classification algorithms has been performed [14]. The prediction of head shaking behavior produced sensitivity and specificity of 75.49% and 99.84%, respectively. The prediction of scratching produced sensitivity and specificity of 80.01% and 99.80%, respectively. False positives were noted in 0.16% of cases for head shaking and 0.20% of cases for scratching. Pet owners were instructed to charge the monitor for a period of 2–3 h once per week on a consistent schedule and to otherwise keep the monitor on the dog at all times, although they were asked to remove the activity monitor if the dog was expected to be in water for an extended period of time.

Activity monitor assessments focused on scratching, head shaking and sleep quality behavioral data, which were identified as representing important aspects of the dogs’ pruritic condition. 

### 2.5. Study Food

Dogs were fed Hill’s Prescription Diet^®^ Derm Defense^TM^ Canine dry food in quantities appropriate for their weight per the feeding guide on the label. The nutrition composition of the study food is shown in Table 2; nutrient levels meet or exceed the Association of American Feed Control Officials’ guidelines for complete and balanced nutrition for maintenance of adult dogs. Veterinarians were permitted to adjust the recommended quantity of food as needed to support optimal weight. Foods were provided in commercially labelled bags and measuring cups were provided to assist in accurately measuring food. The impact of the Derm Defense^TM^ food on skin and coat health and immune modulation in dogs with seasonal atopy or atopic dermatitis has previously been evaluated [15,16].

### 2.6. Study Dismissal

Dogs were eligible for removal from the study under any of the following circumstances: the owner requested to leave the study; the investigator felt that the pet should not continue on study for any reason; the condition was not improved within 28 days (four weeks) after the start of the study food, based on the investigator’s assessment; diagnosis warranted an increase in medications; food intake was less than 60% of recommendation for at least five consecutive days; there was poor owner compliance; the dog required a change to a different food due to intolerance of the study food or diagnosis of a disease requiring a change in food; a life-threatening illness or accident occurred or death or owner-elected euthanasia.

### 2.7. Statistical Analysis

Scratching and head shaking behaviors recorded by the Vetrax^®^ activity monitor were summarized in units of time representing the duration of each behavior (seconds per day). Sleep quality was measured using a proprietary algorithm based on absence of night-time disturbance and scaled 0–100 (0: highly disturbed sleep, 100: undisturbed sleep). Lower values for scratching and head shaking behaviors and higher values for sleep quality are postulated to be associated with improvements in the pruritic condition. These measures were collected continuously and aggregated into daily and per phase metrics. Patterns in daily behaviors for individual dogs were plotted from Day –14 to Day 56. In order to compare activity monitor data to questionnaire data collected at visits on Day 0, Day 28 and Day 56, an overall estimate of study baseline for each activity of interest was established using the mean daily duration of each behavior during the two-week baseline period. 

Analysis of changes in behavior were conducted using standard statistical methods and general linear mixed effects models using the package lme4 [17]; all model estimates were by maximum likelihood and residuals were checked for homogeneity and normality. These models were used to estimate predictive power of study phase (baseline, phase 1 or phase 2) and/or study day on the pruritic indicators as assessed by the veterinarian (overall clinical signs of pruritus and skin and coat quality), pet owner (overall condition of skin and haircoat, disruption to family, pet quality of life) or activity monitor data on mean daily duration of pruritic behaviors (scratching, head shaking and sleep quality). Random effects of clinic and individual were included to account for veterinarian bias and repeated measures, respectively. The effect of potentially confounding variables (age, sex, weight, BFI, spore count, pollen count, humidity, outdoor temperature, precipitation, daylight hours, date and medication use) were assessed by testing each as a predictor of each outcome variable. Where variables were not found to significantly predict study outcomes, they were excluded from further analysis. Ultimately, all models were adjusted for BFI, weight, daylight hours, pollen count, and medication use (Benadryl, Apoquel, hydroxazine). The full dataset used in regression modes can be found in Supplemental Table S3. 

To provide a more tangible insight into the fit of the models, predictions were generated for each pruritic indicator for each phase of the study to represent a hypothetical average study participant. Data used to describe the confounding variables provided to the model were drawn from the distribution of values across all study dogs at each phase of the study. 

In order to account for the possibility of false discovery caused by multiple testing, analyses were adjusted using the Benjamini-Hochberg procedure [18]. An alpha level of significance of false discovery rate (FDR)-adjusted *p* < 0.05 was used throughout; however, because of the small number of dogs, FDR-adjusted *p* < 0.1 were also considered and have been reported. Standardized coefficients have been calculated and reported; in order to provide a more intuitive insight into the data unstandardized coefficients are provided for selected results. All data were preprocessed using MATLAB [19] and analyzed using R [20]. 

## 3. Results

### 3.1. Demographic Characteristics

Demographic information on dogs that completed the study is shown in Table 3; individual dog characteristics are provided in Supplementary Table S1. Seven dogs with pruritus enrolled in the study. The majority of dogs were female, all were spayed or neutered, and the average age was 6.4 ± 2.0 years. All dogs completed the study, however, one dog’s activity monitor data was not useable due to a technical issue; therefore, six dogs were included in this interim analysis.

There were no major protocol violations during the study, only minor deviations. One deviation was a dog residing in a multi-dog household. In several instances, dogs were reported as having clinic visits scheduled outside of the visit window, but the infraction was not considered major. In several instances, scheduling issues resulted in a period of time between the Day –14 and Day 0 visits that was greater than 14 days; however, in all cases the baseline period was defined as the fourteen days immediately prior to the Day 0 visit and any data collected prior to this was discarded. A total of 6 AE were reported during the study, 4 involving the integumentary system (3 mild, 1 moderate in severity) and 2 involving the gastrointestinal system (both mild in severity). The 6 AE were determined to be either not study-related (4) or unlikely to be study-related (2). No severe, life threatening, or fatal AE were reported. 

### 3.2. Food Acceptance and Medication Use

The food was well-accepted by the pets: 100% (6/6) dogs were reported to eat 100% of food offered at Day 28, 83.3% (5/6) dogs were reported to eat 100% of their food offered at Day 56 and the sole dog that did not consume 100% was reported to eat 80% of its food at Day 56. Fecal scores indicated that the food was well-tolerated by study participants. The average fecal score at Day 28 was 4.5 (range 3.9–5.0) and the average fecal score at Day 56 was 4.1 (range 2.4–5.0). 

Pet owners scored food acceptance and food aroma based on a scale of 0 to 100. The average of how well the dogs liked the study food (0: rejected totally, 100: liked extremely) on Day 28 was 91.0 (range 80–100) and 91.2 (range 80–100) on Day 56. Enthusiasm while consuming the study food (0: no enthusiasm, 100: extreme enthusiasm) was a mean of 89.3 (range 70–100) on Day 28 and 90.8 (range 80–100) on Day 56. The strength of the aroma of the study food (0: no aroma, 100: very strong aroma) was an average of 55.0 (range 10–100) on Day 28 and 50.0 (range 15–100) on Day 56, while the pleasantness of the study food aroma (0: not pleasant at all, 100: extremely pleasant) was 68.6 (range 50–100) on Day 28 and 67.7 (range 50–100) on Day 56. Day 28 responses to the last question include only five responses, as one pet owner indicated that they did not observe the aroma. All other responses included all six patients.

Pet owners were asked to report when their dogs consumed food other than the study food. At Day 28, 33.3% (2/6) of pet owners reported at least one instance of their dogs consuming non-study food. At Day 56, 66.7% (4/6) of pet owners reported at least one instance. Non-study foods consumed consisted of occasional table scraps and treats and were not considered significant in quantity.

As permitted in the inclusion criteria, management of AD in these dogs involved multimodal therapies in addition to the therapeutic food. Medications included non-steroidal anti-inflammatory drugs, janus kinase inhibitors, topical treatments, antihistamines, and corticosteroids. Three out of seven pruritus patients (Dogs 3, 5 and 6) decreased or stopped at least one of the medications related to their skin condition. 

### 3.3. Effect of Study Phase on Pruritic Indicators

Nine overall metrics were evaluated as pruritic indicators in this interim analysis, including scratching, head shaking and sleep quality, as measured by the Vetrax^®^ activity monitor, the overall clinical signs of pruritus and skin and coat quality as assessed by the veterinarian and the dog’s quality of life, current level of disruption to the owner’s family caused by the dog’s skin condition and overall condition of skin and haircoat as assessed by the owner. The assessment scales used in rating these indicators, along with the range of reported assessments and mean value of each indicator at each phase of the study, are shown in Table 4. Predicted values for each pruritic indicator for a hypothetical average study participant were similar (Supplementary Table S2). 

A series of mixed effect models were run to predict these pruritic indicators as a function of study phase (Table 5), and study phase was found to be a significant predictor of all of the metrics when accounting for extraneous variables. Scratching and head shaking significantly decreased over time, while sleep quality significantly increased. Overall clinical signs of pruritus and effect on condition of both coat and skin as reported by veterinarians decreased significantly over time. The current level of disruption to the owner’s family caused by the dog’s skin condition significantly decreased over time, while the dog’s quality of life and the overall condition of skin and haircoat as reported by the pet owner significantly improved. Standardized coefficients indicate that the largest changes were seen in the veterinarian assessments of the effect on the dog’s coat quality (β = −1.038, adjusted *p* < 0.001), skin quality (β = −0.517, adjusted *p* < 0.001) and overall clinical signs of pruritus (β = −0.713, adjusted *p* < 0.001). Unstandardized coefficients indicate that for each one unit (four week) increase in study phase, with all other model parameters remaining constant, the veterinarian assessment of the dog’s coat quality improved by an average of 28.13 points on the 100-point visual analogue scale; similarly, the veterinarian assessment of the dog’s skin quality improved by an average of 15.86 points, and veterinarian-assessed overall clinical signs of pruritus improved by an average of 18.49 points.

### 3.4. Effect of Study Day on Pruritic Indicators

**Figure 1:**
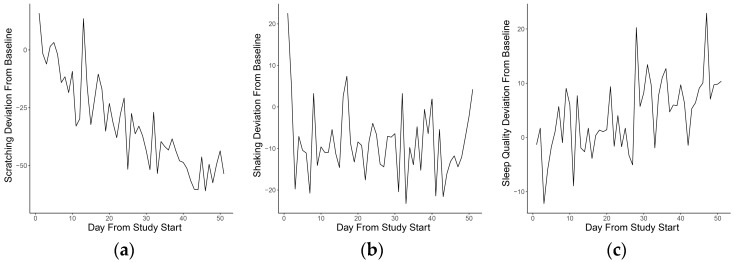
Deviation from baseline of mean daily values for three key behaviors for each day of the study. Points on the vertical axis represent the difference between baseline and daily mean for (**a**) scratching; (**b**) head shaking and (**c**) sleep quality.

Study day was found to significantly predict the amount of scratching and sleep quality, as measured by the Vetrax^®^ activity monitor in adjusted models (Table 6). As the study progressed, scratching significantly decreased (β = −0.339, adjusted *p* = 0.0007) and sleep quality increased (β = 0.247, adjusted *p* = 0.0062). Unstandardized coefficients indicate that for each additional study day, with all other model parameters remaining constant, scratching decreased by 1.43 s and sleep quality improved by 0.275 units. Mean daily values of scratching, shaking and sleep quality recorded during the 56-day study are plotted in Figure 1. 

### 3.5. Effect of Vetrax^®^-Assessed Behaviors on Veterinarian-Assessed Pruritic Indicators

A series of mixed effect models were run to evaluate the predictive effect of 3 Vetrax^®^ metrics on each of three veterinarian-assessed pruritic indicators (Table 7). Scratching was found to significantly predict overall clinical signs (β = 0.176, adjusted *p* = 0.008), indicating that as scratching increased, overall clinical signs worsened. The effect of scratching on overall coat quality and overall skin quality was consistent with the patterns observed for overall clinical signs, but the FDR-adjusted *p* value for overall skin quality did not surpass the pre-specified significance threshold. Head shaking was found to significantly predict overall clinical signs and overall coat quality (β = 0.197, adjusted *p* < 0.001; β = 0.122, adjusted *p* = 0.024). Finally, sleep quality was found to significantly predict overall clinical signs (β = −0.154, adjusted *p* < 0.001) and overall skin quality (β = −0.103, adjusted *p* = 0.032). Standardized coefficients indicate that the largest changes were seen for the predictors of overall clinical signs: head shaking (β = 0.197), scratching (β = 0.176) and sleep quality (β = −0.154). With all other model parameters remaining constant, unstandardized coefficients indicated that for each one s/day increase in head shaking, overall clinical signs worsened by 0.058 units, for each one s/day increase in scratching, overall clinical signs worsened by 0.025 units and for each one unit improvement in sleep quality, overall clinical signs improved by 0.082 units.

### 3.6. Effect of Vetrax^®^-Assessed Behaviors on Owner-Assessed Pruritic Indicators

A series of mixed effect models were run to evaluate the predictive effect of 3 Vetrax^®^ metrics on each of three owner-assessed pruritic indicators (Table 8). Scratching was found to significantly predict quality of life and overall condition of skin and haircoat (β = −0.103, adjusted *p* = 0.013; β = 0.119, adjusted *p* = 0.047), indicating that as scratching increased, quality of life and overall condition of skin and haircoat worsened. Head shaking was found to significantly predict quality of life and overall condition of skin and haircoat (β = −0.146, adjusted *p* < 0.001; β = 0.092, adjusted *p* = 0.043). The effect of head shaking on disturbance to the family was not statistically significant. Finally, sleep quality was not found to significantly predict any of the three owner assessments. Standardized coefficients indicate that the largest changes were seen in head shaking as a predictor of quality of life (β = −0.146), scratching as a predictor of the overall condition of skin and haircoat (β = 0.119), and scratching as a predictor of quality of life (β = −0.103). Unstandardized coefficients indicated that, with all other model parameters remaining constant, for each one s/day increase in head shaking, quality of life worsened by 0.006 units, for each one s/day increase in scratching, the overall condition of skin and haircoat worsened by 0.03 units, and for each one s/day increase in scratching, quality of life worsened by 0.002 units. 

Comparisons of owner assessments to activity monitor measures revealed instances in which the owner may have been unaware of changes in pruritic behaviors over time. For example, the owners of Dog 2 rated ear scratching at similar levels on Days 0, 28 and 56, despite reductions in scratching documented by the Vetrax^®^ activity monitor during the corresponding study phases (Figure S1, panels c and d). Conversely these comparisons also highlighted instances in which the sensor reported little change in some dimensions of pruritic behaviors while the owner questionnaire responses suggested changes had occurred (Figure S1, panels h, k and l corresponding to Dogs 4 and 6) suggesting a mismatch between the subjective reporting and the automated reporting of behaviors. In other instances, the accelerometer and owner reports were in agreement (Figure S1, panels e, f, g, i and j corresponding to Dogs 3, 4 and 5); in panels e, f, i and j the accelerometer and owner both reported decreases in scratching over time while in panel g (corresponding to Dog 4), both the accelerometer and the owner registered a small increase in scratching in Phase 1 compared to baseline, then a decrease in Phase 2. 

## 4. Discussion

The aim of this study was to assess the use of triaxial accelerometer activity monitors as tools for the objective assessment of behavior in dogs with pruritus. The activity monitors were used to measure behavioral indicators that represented important aspects of canine pruritus: scratching, head shaking and sleep quality. Veterinarian and owner questionnaires were completed to provide details of the current state of the condition in the dogs. 

The ability to predict the behaviors measured by the activity monitors and those reported by veterinarians and pet owners as a function of time since initiation of study food (represented by study phase and study day) was assessed using generalized linear mixed effect models fit to the activity monitor data. Scratching and head shaking significantly decreased as a function of study phase while sleep quality significantly increased; results from models using study day as a predictor were consistent with those using study phase. Overall clinical signs of pruritus and effect on condition of both coat and skin as reported by veterinarians decreased significantly as a function of study phase. Similarly, current level of disruption to the owner’s family caused by dog’s skin condition decreased significantly as a function of study phase, while the dog’s quality of life and the overall condition of skin and haircoat as reported by the pet owner improved. Taken together, these models indicated that the pruritus indicators captured by the Vetrax^®^ activity monitor (scratching, head shaking and sleep quality) as well as those reported by the veterinarian and pet owner significantly improved as the duration of time in the study and length of time fed Hill’s Prescription Diet^®^ Derm Defense^TM^ Canine dry food increased. 

The pruritus indicators measured by the Vetrax^®^ activity monitor were found to significantly predict both veterinarian and pet owner assessments. Veterinarian-assessed overall clinical signs of pruritus were significantly predicted by scratching, head shaking and sleep quality, while overall coat quality was significantly predicted by head shaking. Owner-assessed quality of life and overall condition of skin and haircoat were significantly predicted by scratching and head shaking. However, in some cases, apparent mismatches between owner reports of individual behaviors and accelerometer assessments were noted. Such mismatches may occur for a variety of reasons, including the difficulty for owners in performing subjective assessment in a continuous manner over the course of several weeks, the potential for missed observations and misinterpretation of observations by owners, the episodic nature of the behaviors and the pruritic condition itself, or even differences in the individual completing the questionnaire from visit to visit. Furthermore, motions that are captured as a single behavior on the sensor may span multiple items on the owner questionnaire, and changes observed by owners may span multiple behaviors captured by the sensor. For example, the sensor is programmed to report specific instances of a pruritic behavior, such as scratching. However, the owner may observe this behavior as a specific type of scratching (ex., ear or skin scratching) or may observe changes that are potentially related to several pruritic behaviors (such as condition of skin and haircoat, disruption to family, and quality of life). Such reporting issues impact everyday interactions between owners and veterinarians, even apart from clinical studies, highlighting the value of a device that can provide continuous, quantitative, and objective data on the frequency and duration of pruritus-related behaviors. 

Inertial measurement units are established as a predominant approach to measuring movement-based behavior in animals and humans [21,22,23]. Techniques to interpret the movements of the subject in terms of the behaviors exhibited, including signal processing and machine learning strategies, can provide insight into the subject’s behavior that would otherwise involve a significant time investment through manual observation and behavior sampling. In human patients with AD and other pruritic conditions, activity data collected using limb-worn accelerometers (Actiwatch Plus, ActiTrac, Actigraph Motionlogger, DigiTrac) was found to correlate well with video analysis of scratching [24,25]. One study reported a correlation between wrist accelerometer data and the overall and subjective scoring of AD (SCORAD) [26] but most reports failed to find a correlation between accelerometer data and subjective measures of itch intensity [24,27,28,29,30,31]. One study reported a correlation between wrist accelerometer data and clinical assessments of disease severity, extent and intensity as well as AD-associated chemokine markers [31]. Three previous studies in dogs [9,10,32] have evaluated the use of collar-mounted accelerometers for assessment of pruritus. Using Actiwatch^®^ monitors, higher mean activity levels were seen in AD dogs compared to healthy dogs during the day, evening and overnight [9]. Two studies found strong correlations between nocturnal Acti-Cal^®^ accelerometer data and video analysis of scratching [10,32]. In flea-sensitized dogs, the accelerometer also detected differences between baseline and post-challenge periods as well as between prednisolone-treated and untreated groups [32]. Overall, these data show that accelerometers have been used successfully to monitor pruritic activity in groups of dogs, although associations with subjective assessments of itch have not been consistently found. However, the accelerometers used in all of these studies detected general motion and were not able to distinguish specific pruritic behaviors.

The findings reported here suggest opportunities for more advanced management of pruritus in dogs. Because activity monitors are able to capture a detailed, objective and continuous record of the dogs’ behavior, these devices may provide insight into a dog’s condition that would be otherwise unavailable. The role of the veterinarian in diagnosis and treatment, however, remains critical. While the activity monitor is able to supplement the veterinarian’s assessment with an objective behavior record, expert interpretation of the behavior, incorporating clinical observations, laboratory data, knowledge of the animal and information reported by the owner, is irreplaceable in order to ensure an appropriate treatment plan. As described [7], treatment plans for pruritic conditions such as dermatitis are “likely to vary between dogs and, for the same dog, between times when the disease is at different stages.” The use of activity monitor technology has the potential to support this customization, specifically with regard to progression of the disease over time and response to nutritional therapies.

Strengths of the current study include the evaluation of dogs at multiple clinical practices, the inclusion of both veterinarian and pet owner assessments of the dogs’ conditions, use of a baseline data collection period to measure behaviors before study food was provided, and assessment of medication use and quality of life. Limitations of the study include the fixed set of pruritic behaviors currently recognized by the activity monitor behavior processing algorithms. While the device’s battery life supported a substantial wear-time, the activity monitor was removed from the dog weekly for charging, resulting in periods of time of varying length which are missing from the activity monitor data. Furthermore, the presence of the activity monitor on the collar may have altered the expression of certain behaviors in the dogs involved in the study. Use of outcome assessment tools specific to pruritic conditions such as dermatitis, for example, a disease-specific quality of life instrument [33,34,35] or a validated pruritus scoring system, such as the canine pruritus severity scale [8,14], may have increased the power of the study, since they would have reduced outcome misclassification. A longer study may have been necessary to detect changes in medication use given the episodic nature of pruritus. Activities determined by the pet owner, such as the amount of time each dog spent outdoors, could have influenced exposure to environmental allergens. These activities were considered in statistical models but were not controlled in the study design. Finally, being overweight or obese is a well-recognized and common problem in companion animals; here dogs gained an average of 7% of their weight at study entry by study Day 56. More detailed guidance on achieving and maintaining a desirable weight may have helped pet owners avoid weight gain in their pets.

Future refinements to sensing technology and behavior algorithms as well as the identification of behavior patterns that characterize healthy and diseased animals may increase the usefulness of sensing technology in making the initial diagnosis of a medical condition and enabling more sophisticated clinical management. 

## 5. Conclusions

Wearable sensor technology is an exciting new frontier that offers tremendous benefits in veterinary medicine. The use of sensing technology for canine pruritus management has undergone significant innovation from simply scoring pruritus via increased overall activity to objective assessment of specific pruritic behaviors. In this interim data analysis, the pruritic behaviors measured using the Vetrax^®^ wearable sensor technology were strongly correlated with veterinary clinical and pet owner assessments. With additional study enrollment, a fully powered evaluation of veterinarian and pet owner assessments can be completed, including a more detailed consideration of changes in medication use and evaluation of pruritic behaviors. The data available to date suggest that the Vetrax^®^ sensing technology can be an effective tool for objectively documenting the response to nutritional therapies for pruritus.

## Figures and Tables

**Table 1:** Timing of study assessments.

	Day –14	Day 0	Day 28	Day 56
**Consent and Release Forms**				
Informed Consent	x			
Model Talent Consent and Release	x			
Animal Talent Consent and Release	x			
**Clinical Report Forms**				
Eligibility Checklist (Part I)	x			
Eligibility Checklist (Part II)		x		
Medical History	x			
Medication Record		x	x	x
Dietary History	x			
Physical Exam	x	x	x	x
Veterinary Clinical Evaluation		x	x	x
Pet Owner Evaluation		x	x	x
Pet Owner Food Questionnaire			x	x

**Table 2:** Nutritional composition of study food.

Item ^1^	Hill’s Prescription Diet^®^ Derm Defense^TM^ Canine Dry Food
Protein, %	23
Fat, %	15
Crude Fiber, %	1.6
Nitrogen-free Extract, %	55
Ash, %	5.7
Calcium, %	1.0
Phosphorous, %	0.8
Sodium, %	0.3
Potassium, %	0.8
Magnesium, %	0.11
Vitamin C, ppm	151
Vitamin E, IU/kg	823
Total Omega-3 Fatty Acids, %	1.8
Total Omega-6 Fatty Acids, %	4.0
Metabolizable Energy, kcal/kg	3948

^1^ All nutrients are expressed on a 100% dry matter basis unless otherwise noted. IU: International Units; ppm: parts per million.

**Table 3:** Demographics of privately owned dogs with pruritus.

Characteristic	
N	7
Age in yrs., mean (range)	6.4 (3.1–8.4)
Female, %	57.1
Spayed/neutered, %	100
Mixed breed, %	42.9
Weight at Day 0 in kg, mean (range)	20.4 (9.5–32.0)
Weight at Day 28 in kg, mean (range)	21.3 (10.5–34.5)
Weight at Day 56 in kg, mean (range)	21.8 (10.4–36.3)
Mean weight change (Day 0 to Day 56), %	+7.0
BFI at Day 0, mean (range)	27.1 (20–40)
BFI at Day 28, mean (range)	30.0 (20–40)
BFI at Day 56, mean (range)	30.0 (30–40)
Mean BFI change (Day 0 to Day 56), %	+10.5

BFI: Body Fat Index (scored on a visual scale of 20–70).

**Table 4:** Pruritic indicators by study phase.

Pruritic Indicator	Assessment Scale ^1^	Assessed By	Range	Baseline ^2^ Mean ± SE	Phase 1 ^2^ Mean ± SE	Phase 2 ^2^ Mean ± SE
Scratching (s/day)	N/A	Vetrax^®^	0–459	96.87 ± 11.40	65.82 ± 4.86	34.21 ± 1.91
Head Shaking (s/day)	N/A		0–297	49.15 ± 5.47	45.82 ± 2.14	46.20 ± 2.41
Sleep Quality	0: Highly disturbed; 100: Undisturbed		4–100	47.13 ± 1.78	50.90 ± 1.49	59.53 ± 1.85
Overall Clinical Signs	0: No signs; 100: Severe signs	Veterinarian	0–41	22.61 ± 1.08	15.40 ± 0.71	7.45 ± 0.54
Overall Skin Quality	0: Excellent; 100: Poor		0–41	24.46 ± 1.06	16.69 ± 0.97	4.37 ± 0.28
Overall Coat Quality	0: Excellent; 100: Poor		0–41	26.53 ± 1.23	9.82 ± 0.33	2.08 ± 0.13
Quality of Life	1: Very Poor; 10: Excellent	Pet Owner	4–10	6.49 ± 0.16	7.49 ± 0.12	7.78 ± 0.08
Current Level of Disruption to Family Caused by Dog’s Skin Condition	0: Not disruptive; 100: Extremely disruptive		0–70	38.52 ± 2.00	24.97 ± 1.36	19.34 ± 1.88
Overall Condition of Skin and Haircoat	0: Very healthy; 100: Extremely poor		0–67	44.05 ± 1.20	36.81 ± 1.49	16.67 ± 0.73

^1^ Pruritic indicators assessed by veterinarians and pet owners were rated on a visual analog scale with the indicated assessment scale range. Lower numbers are associated with improved condition for all metrics except sleep quality and quality of life; ^2^ Baseline: Days –14 to 0; Phase 1: Days 1 to 28; Phase 2; Days 29 to 56. SE: standard error.

**Table 5:** Effect of study phase on pruritic indicators.

Pruritic Indicator	β Coefficient ^1^	*p* Value ^2^
Scratching ^3^	−0.173	0.0413
Head Shaking ^3^	−0.226	0.0105
Sleep Quality ^3^	0.208	0.0437
Overall Clinical Signs	−0.713	< 0.001
Overall Skin Quality	−0.517	< 0.001
Overall Coat Quality	−1.038	< 0.001
Quality of Life	0.291	< 0.001
Disruption to Family	−0.387	< 0.001
Overall Condition of Skin and Haircoat	−0.401	< 0.001

^1^ All models adjusted for Body Fat Index, weight, daylight hours, pollen count, medication use and clinic and controlled for repeat measures; ^2^ *p* values adjusted for false discovery rate using the Benjamini-Hochberg procedure; ^3^ Activity monitor data was analyzed as mean daily duration of each behavior averaged over each study phase. Lower numbers are associated with improved condition for all metrics except sleep quality and quality of life.

**Table 6:** Effect of study day on pruritic indicators.

Pruritic Indicator	β Coefficient ^1^	*p* Value ^2^
Scratching ^3^	−0.339	0.0007
Head Shaking ^3^	−0.224	0.0801
Sleep Quality ^3^	0.247	0.0062

^1^ All models adjusted for Body Fat Index, weight, daylight hours, pollen count, medication use and clinic and controlled for repeat measures; ^2^ *p* values adjusted for false discovery rate using the Benjamini-Hochberg procedure; ^3^ Activity monitor data was analyzed as mean daily duration of each behavior.

**Table 7:** Effect of activity monitor measures on veterinarian assessments. ^1^

Pruritic Indicator: Predictor ^1^	Pruritic Indicator: Outcome	β Coefficient ^1^	*p* Value ^2^
Scratching	Overall Clinical Signs	0.176	0.008
	Overall Skin Quality	0.083	0.263
	Overall Coat Quality	0.125	0.099
Head Shaking	Overall Clinical Signs	0.197	<0.001
	Overall Skin Quality	−0.012	0.884
	Overall Coat Quality	0.122	0.024
Sleep Quality	Overall Clinical Signs	−0.154	<0.001
	Overall Skin Quality	−0.103	0.032
	Overall Coat Quality	−0.085	0.085

^1^ Regressions were performed between mean daily values from each activity monitor measure and veterinarian-assessed pruritus clinical signs; all models adjusted for Body Fat Index, weight, daylight hours, pollen count, medication use and clinic and controlled for repeat measures; ^2^ *p* values adjusted for false discovery rate using the Benjamini-Hochberg procedure.

**Table 8:** Effect of activity monitor measures on owner assessments. ^1^

Pruritic Indicator: Predictor ^1^	Pruritic Indicator: Outcome	β Coefficient ^1^	*p* Value ^2^
Scratching	Quality of Life	−0.103	0.013
	Disruption to Family	−0.029	0.415
	Overall Condition of Skin and Haircoat	0.119	0.047
Head Shaking	Quality of Life	−0.146	<0.001
	Disruption to Family	0.002	0.987
	Overall Condition of Skin and Haircoat	0.092	0.043
Sleep Quality	Quality of Life	0.027	0.328
	Disruption to Family	−0.020	0.394
	Overall Condition of Skin and Haircoat	−0.038	0.391

^1^ Regressions were performed between mean daily values from each activity monitor measure and owner-assessed pruritus signs; all models adjusted for Body Fat Index, weight, daylight hours, pollen count, medication use and clinic and controlled for repeat measures; ^2^ *p*-values adjusted for false discovery rate using the Benjamini-Hochberg procedure.

## Supplementary Materials

The following are available online at http://www.mdpi.com/1424-8220/18/1/249/s1, Table S1: Individual dog characteristics; Table S2: Predicted expression of pruritic indicators using models fit to study data; Table S3: Data file; Figure S1: Mean duration scratching per phase versus owner-assessed ear scratching for (**a**) dogs 1, (**c**) 2, (**e**) 3, (**g**) 4, (**i**) 5, and (**k**) 6 and skin scratching for (**b**) dogs 1, (**d**) 2, (**f**) 3, (**h**) 4, (**j**) 5, and (**l**) 6.
