# Supplementary material for: Use of Accelerometer Activity Monitors to Detect Changes in Pruritic Behaviors: Interim Clinical Data on 6 Dogs
Source: Sensors (Basel). 2018 Jan 16;18(1):249. doi: 10.3390/s18010249 (PMC5795410; doi:10.3390/s18010249)
Supplement: Supplementary file 1 [file sensors-18-00249-s001.zip › sensors-253121-supplementary/Tables S1,S2.docx]

Article

Use of Accelerometer Activity Monitors to Detect Changes in Pruritic Behaviors: Interim Clinical Data on 6 Dogs

Susan M. Wernimont ^1^*, Robin J. Thompson ^2^, Scott L. Mickelsen ^3^, Spencer C. Smith ^4^, Isabella C. Alvarenga ^5^, and Kathy L. Gross ^6^

^1^ Pet Nutrition Center, Hill’s Pet Nutrition, Inc., Topeka, KS 66617, USA; [sue_wernimont@hillspet.com](mailto:sue_wernimont@hillspet.com)

^2^ Open Lab, Newcastle University, Newcastle-upon-Tyne, UK; r.j.thompson3@newcastle.ac.uk

^3^ Pet Nutrition Center, Hill’s Pet Nutrition, Inc., Topeka, KS 66617, USA; [scott_mickelsen@hillspet.com](mailto:scott_mickelsen@hillspet.com)

^4^ Department of Grain Science and Industry, Kansas State University, Manhattan, KS 66506, USA; [spence5@k-state.edu](mailto:spence5@k-state.edu)

^5^ Department of Grain Science and Industry, Kansas State University, Manhattan, KS 66506, USA; [isacorsato@ksu.edu](mailto:isacorsato@ksu.edu)

^6^ Pet Nutrition Center, Hill’s Pet Nutrition, Inc., Topeka, KS 66617, USA; [kathy_gross@hillspet.com](mailto:kathy_gross@hillspet.com)

***** Correspondence: sue_wernimont@hillspet.com; Tel.: +01-785-286-8157

**Table S1.** Individual dog characteristics.

| **Patient** | **Breed** | **Sex** | **Age (years)** | **Day 0**  **Weight (kg)** | **Day 0**  **BFI** | **Day 0**  **Visit Date** |
| --- | --- | --- | --- | --- | --- | --- |
| Dog 1 | Labrador / Pit Bull Mix | Male / Neutered | 4.1 | 20.1 | 20 | 3/2/2017 |
| Dog 2 | King Charles Spaniel | Female / Spayed | 8.4 | 9.5 | 30 | 11/30/2016 |
| Dog 3 | Terrier Mix | Female / Spayed | 3.1 | 18.0 | 30 | 11/23/2016 |
| Dog 4 | Labrador | Male / Neutered | 7.3 | 32.0 | 20 | 12/1/2016 |
| Dog 5 | Boxer | Female / Spayed | 6.4 | 22.7 | 30 | 11/23/2016 |
| Dog 6 | Labrador Mix | Male / Neutered | 7.9 | 29.4 | 40 | 11/22/2016 |
| Dog 7 ^1^ | Miniature Schnauzer | Female / Spayed | 7.6 | 11.0 | 20 | 1/2/2017 |

^1^ Not included in interim analysis.^.^ BFI: Body Fat Index.

**Table S2.** Predicted expression of pruritic indicators using models fit to study data.

| **Pruritic Indicator** | **Baseline ^1^** | **Phase 1 ^1^** | **Phase 2 ^1^** |
| --- | --- | --- | --- |
| Scratching | 61.74 | 58.26 | 29.04 |
| Head Shaking | 40.8 | 47.28 | 47.74 |
| Sleep Quality | 46.83 | 50.81 | 59.72 |
| Overall Clinical Signs | 21.77 | 15.71 | 7.79 |
| Overall Skin Quality | 24.1 | 17.69 | 4.75 |
| Overall Coat Quality | 22.76 | 10.8 | 2.78 |
| Quality of Life | 3.33 | 2.28 | 2.08 |
| Disruption to Family | 32.01 | 23.75 | 19.05 |
| Overall Condition of Skin and Haircoat | 44.83 | 36.8 | 16.5 |

^1^ Baseline: Days –14 to 0; Phase 1: Days 1 to 28; Phase 2: Days 29 to 56.
