# Supplementary figures and images for: Use of Accelerometer Activity Monitors to Detect Changes in Pruritic Behaviors: Interim Clinical Data on 6 Dogs
Source: Sensors (Basel). 2018 Jan 16;18(1):249. doi: 10.3390/s18010249 (PMC5795410; doi:10.3390/s18010249)

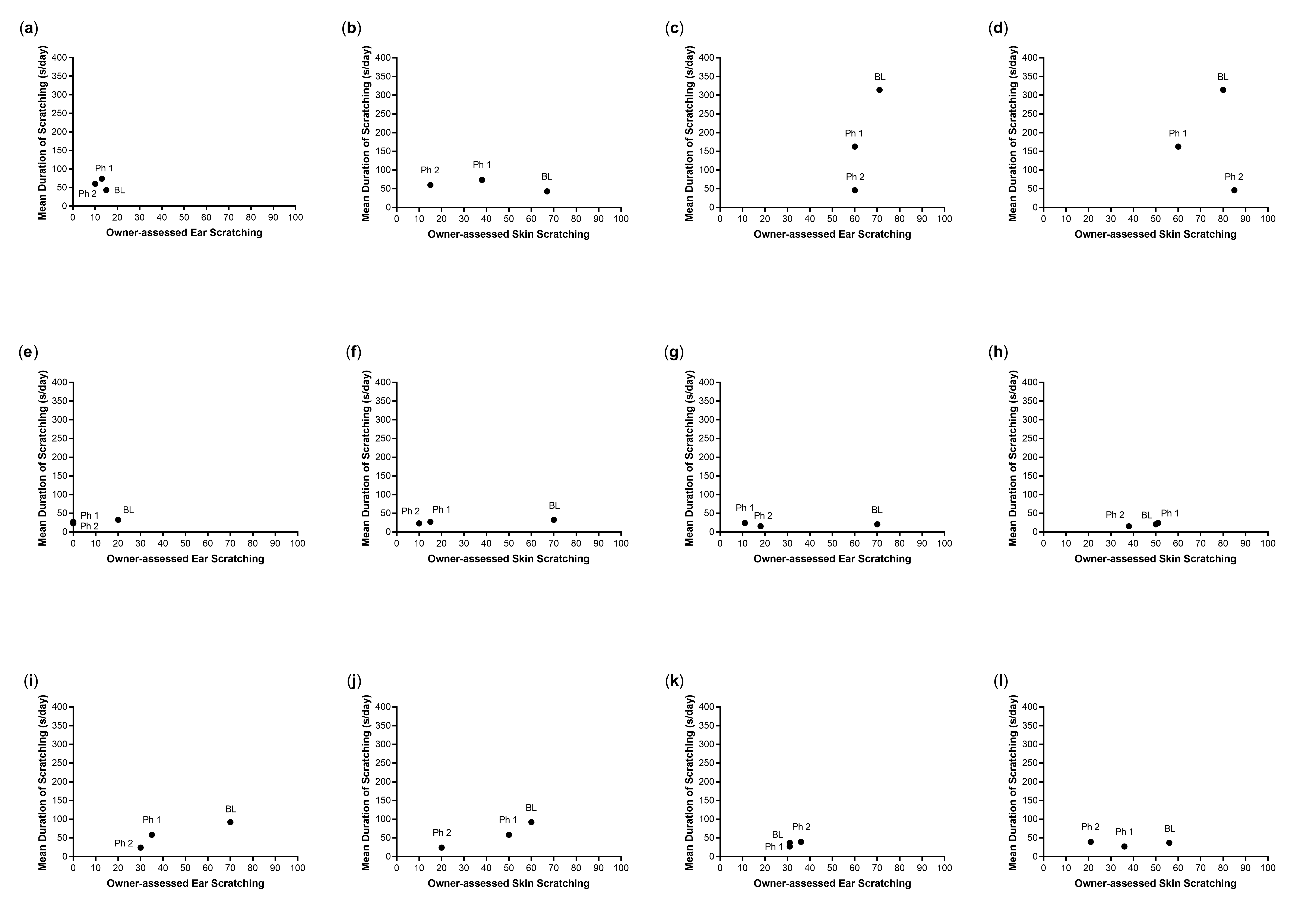

Supplement: Supplementary file 1 [file sensors-18-00249-s001.zip › sensors-253121-supplementary/Figure S1.tif]
